# Presence of clone-specific markers at birth in children with acute lymphoblastic leukaemia

**DOI:** 10.1038/sj.bjc.6600601

**Published:** 2002-10-21

**Authors:** L L Hjalgrim, H O Madsen, M Melbye, P Jørgensen, M Christiansen, M T Andersen, N Pallisgaard, P Hokland, N Clausen, L P Ryder, K Schmiegelow, H Hjalgrim

**Affiliations:** Department of Epidemiology Research, Danish Epidemiology Science Centre, Statens Serum Institut, Artillerivej 5, DK- 2300 Copenhagen S, Denmark; Tissue Typing Laboratory, Department of Clinical Immunology, National University Hospital, Tagensvej 20, DK-2200 Copenhagen N, Denmark; Department of Molecular and Structural Biology, University of Aarhus, C.F Møllers Allé 130, DK-8000 Aarhus C, Denmark; Department of Clinical Biochemistry, Statens Serum Institut, Artillerivej 5, DK-2300 Copenhagen S, Denmark; Department of Haematology and Medicine, Aarhus University Hospital, Tage Hansens Gade 2, DK-8000 Aarhus C, Denmark; Department of Paediatrics, University Hospital of Aarhus at Skejby, Brendstrupgårdsvej 100, DK-8200 Aarhus N, Denmark; Paediatric Clinic II, Juliane Marie Centre, National University Hospital, Blegdamsvej 9, DK-2100 Copenhagen Ø, Denmark

**Keywords:** leukaemia, prenatal origin, TEL-AML 1 fusion gene, tumour burden

## Abstract

Recent studies have suggested that development of childhood acute lymphoblastic leukaemia may often be initiated *in utero*. To provide further evidence of an prenatal origin of childhood leukaemia, we conducted a molecular biological investigation of nine children with B-precursor acute lymphoblastic leukaemia carrying the chromosomal translocation t(12;21), the most common subtype of all childhood acute lymphoblastic leukaemia. Specifically, for each child we identified the non-constitutive chromosomal sequences made up by the t(12;21) fusion gene. From these, leukaemia clone-specific DNA primers were constructed and applied in nested polymerase chain reaction analyses of DNA extracted from the patients' Guthrie cards obtained at birth. Leukaemia clone-specific fusion gene regions were demonstrated in Guthrie card DNA of three patients, age 2 years 11 months, 3 years 4 months, and 5 years 8 months at leukaemia diagnosis. Our findings are consistent with previous observations, and thus provide further evidence that the development of t(12;21) B-precursor acute lymphoblastic leukaemia may be initiated *in utero*. Review of the current literature moreover indicates that age at leukaemia may be inversely correlated with the burden of cells with leukaemia clonal markers, i.e. leukaemia predisposed cells at birth, and that certain types of childhood acute lymphoblastic leukaemia develop as a multiple step process involving both pre- and postnatal genetic events.

*British Journal of Cancer* (2002) **87**, 994–999. doi:10.1038/sj.bjc.6600601 www.bjcancer.com

© 2002 Cancer Research UK

## 

Acute lymphoblastic leukaemia (ALL) is the most common of all childhood cancers. It constitutes a group of different disease entities, which may be characterised by their epidemiology, chromosomal, and molecular genetic aberrations besides by differences in clinical outcome (Greaves, 2002). Few risk factors for ALL have been firmly established and consequently, until recently, little was known about the time or age windows during which genetic events critical to ALL development take place (Greaves, 2002). However, within the last few years solid evidence has accumulated that the first or initiating genetic event in leukaemia development often occurs *in utero*. Specifically, by analysing blood samples obtained at birth in connection with routine screening for metabolic conditions, it has been possible to demonstrate the presence of leukaemia-clone-specific, non-constitutive chromosomal translocations at birth in children diagnosed with leukaemia at the age of several years (Gale *et al*, 1997; Wiemels *et al*, 1999a, 2002).

B-precursor ALL is the most common subtype of childhood leukaemia, and accounts for the peak in ALL incidence seen between the age of 2 and 5 years in the industrialised countries (Greaves, 1999). Nearly 25% of all B-precursor ALL are characterised by a translocation between chromosomes 12 and 21, i.e. t(12;21) (Romana *et al*, 1995). This translocation fuses two putative transcriptional regulators, TEL and AML1, which are both critical to the early haematopoiesis, producing a TEL-AML1 transcript (Rubnitz and Look, 1998). The preponderance and homogeneity of t(12;21) B-precursor ALL make it a good model to study the biology of childhood leukaemia. The literature being limited to one previous study of nine singletons (Wiemels *et al*, 1999a), we therefore found it of interest to study the foetal origin of t(12;21) B-precursor ALL by analysing peri-natally obtained blood samples (Guthrie cards) for the presence of TEL-AML1 fusion genes in a series of patients diagnosed with this specific leukaemia subtype.

## MATERIALS AND METHODS

### Patients and biological samples

In Denmark, neonatal screening for phenylketonuria and congenital myxedema through biochemical analysis of Guthrie cards has been offered since 1980 (Simonsen *et al*, 1998). After analysis, the Guthrie cards with residual blood spots are routinely stored at Statens Serum Institut, Copenhagen. Taking advantage of this unique population-based biological bank, we studied the antenatal origin of childhood leukaemia. Using the population-based Danish Cancer Registry (Storm *et al*, 1997), we identified all children diagnosed with ALL from January 1, 1996, until February 1999, in total 107 patients. Since 1996, reverse transcriptase polymerase chain reactions (PCR) have routinely been performed on diagnostic specimens for all children in Denmark diagnosed with ALL for the presence of the most common genetic aberrations, including t(12;21). We restricted the study population to children with ALL carrying t(12;21) from whom diagnostic tumour material was available, reducing the number of eligible children to 22. Guthrie cards were available for all 22 children. This investigation was approved by the Danish Data Protection Agency (1998-1200-586) and the Scientific Ethics Committee for the Copenhagen and Frederiksberg Municipalities (01-94/98).

### TEL-AML1 gene sequencing used for primer construction

The nucleotide sequences of the TEL-AML1 and the reciprocal AML1-TEL fusion gene regions were established by long distance inverse PCR (LDI-PCR) and sequence determination, as described in detail elsewhere (Andersen *et al*, 2001), using as template high molecular weight chromosomal DNA from leukaemic cells isolated at time of diagnosis. We successfully identified the TEL-AML1 breakpoint region for nine out of 22 patients investigated (Andersen *et al*, 2001). For each of the nine patients, two sets of nested or semi-nested primers encompassing the fusion region were generated and used in a nested PCR reaction to amplify the fusion region directly from the patients' Guthrie cards (Andersen *et al*, 2001). As a prelude to Guthrie card analyses, the specificity of the patient-specific primers was tested in analyses of DNA extracted from corresponding diagnostic tissue samples from the patients (data not shown).

### Guthrie card analysis

Three blood spots, each measuring approximately 1 cm in diameter, are routinely obtained on the Guthrie card from all newborn children. Normally, one or two spots are used for screening purposes, and the residual blood spot(s) are subsequently stored at −20°C. In the present study, two blood spots were available in three and one spot in six out of the nine children included. Genomic DNA was extracted from the Guthrie cards using a QIAamp DNA Mini Kit (QIAGEN, Ltd, UK), the extraction procedure yielding approximately 120 μL DNA extract per blood spot.

As a general control for the integrity or amplificability of blood spot DNA, we performed ‘real time’ PCR on DNA isolated from randomly selected Guthrie card blood spots of 20 children with B-precursor ALL without known chromosomal translocations as well as from 17 healthy children. We used a single-copy gene as target, i.e. the mannose-binding lectin (MBL) gene, located on chromosome 10 (PCR primers: 5′-TGGCAGCGTCTT-ACTCAGAA–3′ and 5′-ATCACTGCA-GGGCAGGTC-3′; TaqMan probe: 5′-6-FAM-CTGTGACCTGTGAGGATGCCCAA –TAMRA-3′, standard set-up and 50 PCR cycles).

For each of the nine children with ALL, a DNA segment covering the break point region was cloned into pBluescript or λ and used to optimise a nested hot start PCR procedure. A dilution range of cloned DNA, from 10^−3^ to 10^−12^ μg μL^−1^, was made in water with salmon sperm (1 : 10 000). Specific primers for a nested procedure were identified to give a final amplicon of 190–300 base pairs. The sensitivity of the PCR procedure was optimised by varying the MgCl_2_ concentration, the number of cycles and the annealing temperature. Optimisation was considered complete when 1–3 copies of the DNA segment could be identified per reaction. Preliminary dilution experiments with DNA extracted from artificially produced Guthrie cards with cloned DNA did not give results different from experiments with cloned DNA diluted in water and salmon sperm (data not shown).

**Figure 1:**
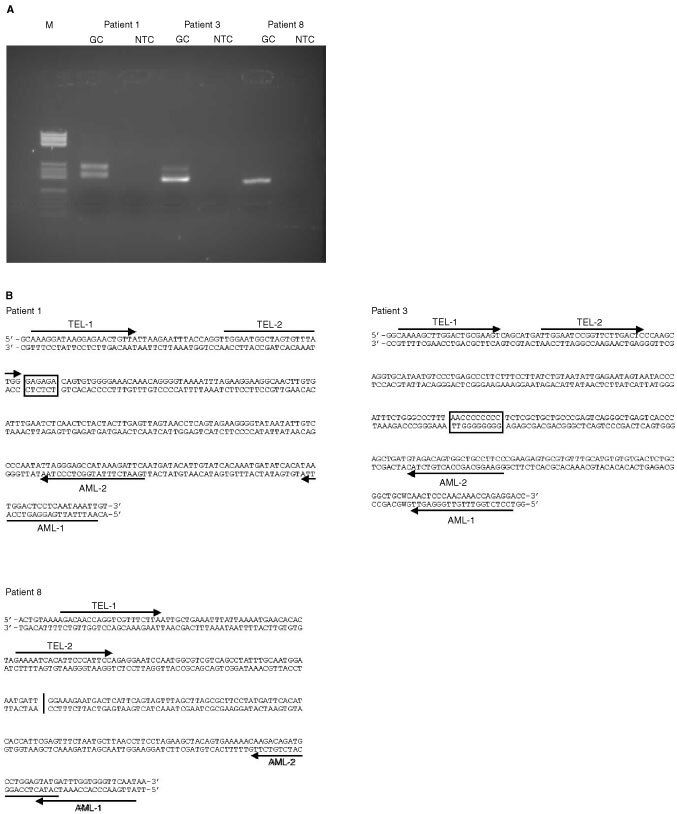
(**A**) Demonstrates the three translocation positive samples run on a 3% agarose gel electrophoresis. **M**: Molecular size marker pBR 327/Hae III. **GC**: DNA extract from the patient's Guthrie card. **NTC**: Non-template control. Sizes of the PCR products were: (Patient 1) 242/190 bp, (Patient 3) 183 bp and (Patient 8) 166 bp. The two fragment sizes in Patient 1, is due to inefficient nesting of one of the second round PCR primers. The identities were verified by direct sequence analysis. (**B**) The three patients individual chromosomal fusion regions are demonstrated. The arrows indicate the PCR primers used in the nested PCR reactions for the detection of the fusion gene (primer 1 followed by primer 2). The vertical line/box shows the site of fusion gene and the box demonstrates the base pairs introduced by the fusion gene event.

Extracted DNA from the nine patients' Guthrie cards was analysed by nested PCR using the conditions obtained by the optimisation analysis. Analyses were performed on successive 10 μL DNA extract volumes until a specific PCR product was obtained or, alternatively, the entire sample of extracted DNA was analysed. A no-DNA control was included in each PCR set-up. Any product of expected size produced by the filter paper PCR was analysed by automated DNA sequencing (ABI 310, Applied Biosystems) either by direct sequencing using an internal primer, or by sequencing of cloned PCR product, using TOPO-TA-cloning Kit (Invitrogen). The sequences of the positive samples were verified by at least two independent sequencing reactions/clones (Figure 1

).

To avoid contamination and prevent false positive results, the fusion-gene sequencing, Guthrie card DNA extraction and optimisation and the final PCR analyses were each carried out at different institutions.

## RESULTS

Twenty-two children with B-precursor ALL and t(12;21) were included in the study. We were able to identify and sequence the TEL-AML1 breakpoint region for nine (41%) of the 22 patients. The success rate was affected by sparse/sub-optimal quality of the diagnostic specimens (primarily low number of leukaemia cells), resulting in low quality and quantity of DNA to be used for determination of the individual breakpoint fusion sequences. The nine patients were between 2 years and 11 months and 6 years and 2 months at leukaemia diagnosis. Leukaemia clone-specific fusion gene sequences were demonstrated in Guthrie card DNA and verified by sequencing in three out of nine patients tested, diagnosed with leukaemia at ages 2 years 11 months, 3 years 4 months, and 5 years 8 months, respectively (Table 1

**Table 1:**
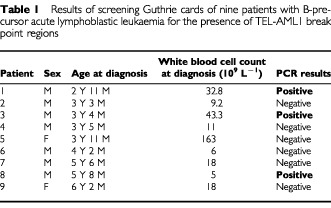
Results of screening Guthrie cards of nine patients with B-precursor acute lymphoblastic leukaemia for the presence of TEL-AML1 break point regions

, Figure 1). The fusion-genes were demonstrated after analysis of 100, 10, and 50 μL DNA extracts, respectively, in consecutively PCR analyses of 10 μL DNA extract each. For the Guthrie cards without evidence of fusion genes, the number of consecutive PCR analyses of 10 μL DNA extract varied from 11 to 21, depending on the number of blood spots available, i.e. one or two. There was no correlation between age of the Guthrie card (data not shown) or tumour burden at diagnosis (Table 1) and outcome of screening the patients' Guthrie cards.

The MBL gene was amplified in all 37 randomly selected Guthrie cards. However, considerable variation in amount of extracted DNA content was observed, ranging from 100 to 8400 (median 760) genome equivalents per 10 μL DNA extract, the volume of DNA extract analysed in the patient-specific translocation analyses. Age of the Guthrie card did not influence the amount of DNA retrieved (data not shown).

## DISCUSSION

We demonstrated the TEL-AML1 fusion genes in three out of nine patients' Guthrie cards in the present investigation. Our findings are compatible with those of Wiemels *et al* (1999a), who demonstrated TEL-AML1 fusion genes in six out of nine patients investigated, and provide further evidence of a prenatal initiation of t(12;21) B-precursor ALL.

Several factors influence the ability to demonstrate leukaemia related genetic markers in DNA extracted from neonatal blood spots. These include the concentration of ‘pre-leukaemic’ cells at birth, the number of cells captured on the filter paper, the state of preservation of the DNA on the filter paper, and the sensitivity of the PCR analyses. Accordingly, while a positive PCR analysis with a high degree of certainty implies the presence of the investigated marker, negative results are less easily interpreted. In the present study the sensitivity of the PCR reactions makes it most likely that negative results are due to absence of translocation specific DNA, i.e. a very low concentration of translocation bearing cells, variations in the effectiveness of the DNA extraction procedure or that the DNA is not amplified sufficiently. The large variation in MBL gene copies recovered from control Guthrie cards indicates limitations in the DNA quality or the DNA extraction procedure. In our experience the yield varies considerably, necessitating an optimisation for each Guthrie card. No single parameter, e.g. the age of the Guthrie card, can be used to predict the effectiveness of extraction. DNA is stable in dried blood spots (Mccabe *et al*, 1987) and in our experience, Guthrie cards can be used as source for DNA at least following storage for 10–15 years at −20°C. In general all cards retrieved at Statens Serum Institut are stored at room temperature for 3 to 5 days before freezing. In order not to reduce the sensitivity of the overall detection of translocation specific DNA, we did not use any of the DNA from the patient-specific Guthrie card as control. Based on the large variation in Guthrie card quality and the fact that the number of TEL-AML1 copies per Guthrie card is Poisson distributed (Larsen *et al*, 1996), we decided not to give an estimation of the number of cells carrying the TEL-AML1 fusion gene at time of birth in case of a positive Guthrie card.

Including the present investigation, Guthrie card analyses have now been reported for a total of 51 singletons with various ALL subtypes in studies employing different types of clonotypic markers (Gale *et al*, 1997; Wiemels *et al*, 1999a; Fasching *et al*, 2000; Yagi *et al*, 2000; Panzer-Grumayer *et al*, 2002; Taub *et al*, 2002) (Tables 2

**Table 2:**
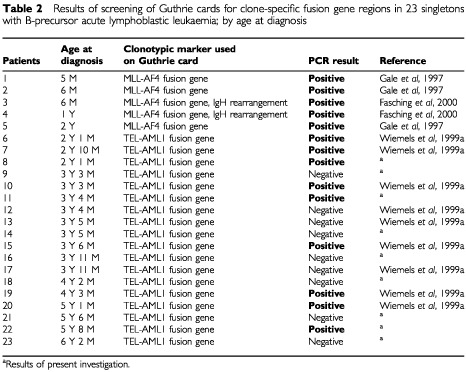
Results of screening of Guthrie cards for clone-specific fusion gene regions in 23 singletons with B-precursor acute lymphoblastic leukaemia; by age at diagnosis

and 3

**Table 3:**
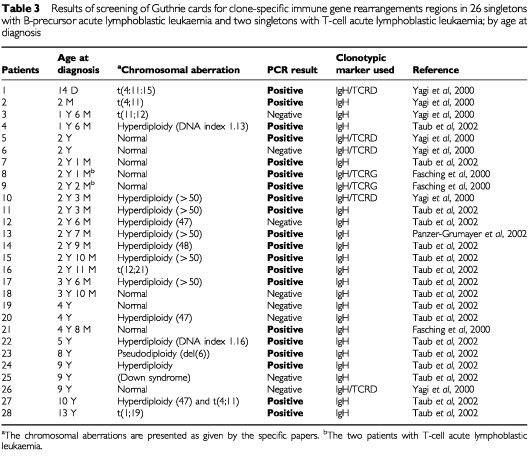
Results of screening of Guthrie cards for clone-specific immune gene rearrangements regions in 26 singletons with B-precursor acute lymphoblastic leukaemia and two singletons with T-cell acute lymphoblastic leukaemia; by age at diagnosis

) and 10 singletons with acute myeloid leukaemia (AML) (Wiemels *et al*, 2002). Despite sharing a prenatal origin with ALL, AML represents a different group of diseases with different natural histories. Accordingly, the Guthrie card results for AML patients will not be discussed further.

Overall, leukaemia markers have been demonstrated in the neonatal blood spots in 34 (66%) of the 51 studied children with ALL. Assuming that all used markers are representative of pre-leukaemic clones, the likelihood of demonstrating the leukaemia markers appears to vary with age at leukaemia diagnosis. Accordingly, Guthrie card analyses have been positive in 88% (seven out of eight) of children aged 0–1 year, in 67% (18 out of 27) of children aged 2–3 years and in 56% (nine out of 16) of children aged 4+ years at leukaemia diagnosis. The inverse correlation between chance of positive PCR result in neonatal Guthrie card analyses and age at diagnosis (Mann–Whitney *P*-value=0.04) may indicate that the burden of cells with the leukaemia clone characteristics at birth is somehow related to age at leukaemia diagnosis. Consistent with this speculation, a recent investigation demonstrated that the number of cells with leukaemia-specific IgH gene rearrangements present in individual Guthrie cards was inversely correlated to age at diagnosis in a series of 12 patients with B-precursor ALL (Taub *et al*, 2002).

Unlike the fusion genes resulting from t(12;21) and t(4;11) used in Guthrie cards analyses, which both appear to be directly involved in the development of leukaemia (Greaves, 2002), this is not the case for the other types of clonal markers used to assess a prenatal origin of leukaemia, TcR and IgH gene rearrangements. Accordingly, whereas the demonstration of t(12;21) and t(4;11) at birth implies the presence of leukaemic or leukaemia-predisposed cells, strictly speaking, no direct inference as to genetic events critical to leukaemia development can be made from the presence of leukaemia-specific TcR and IgH gene rearrangements (Taub *et al*, 2002). Still, neonatal presence of leukaemia clonal markers have been reported in similar proportions of patients studied with t(12;21) or t(4;11) markers and TcR or IgH markers (Tables 2 and 3).

Whereas the wide variety of leukaemia subtypes for which clonal markers have been demonstrated in neonatal blood spots might indicate that foetal initiation is a common phenomenon in childhood leukemia, it is less certain to what extent the presence of cells with leukaemia-specific markers in Guthrie cards reflects complete or incomplete leukaemia development. Leukaemia diagnosed in infants often display t(4;11), and moreover the concordance rate for infant leukaemia is seemingly close to 100% in monochorionic monozygotic twins (Gale *et al*, 1997). Therefore, the demonstration of cells with t(4;11) fusions genes in Guthrie cards from five out of five children examined developing leukaemia before or at the age of 2 years indicate a substantial concentration of t(4;11) carrying cells and consequently that the development of this particular type of leukaemia may already be fully or nearly completed at birth (Table 2). In comparison, the later occurrence of t(12;21) B-precursor ALL, i.e. typically at ages 2–5 years, in combination with the demonstration of t(12;21) carrying cells in Guthrie cards in children diagnosed with leukaemia at the age of 5 years in the setting of a generally less frequent demonstration of leukaemia clone-specific t(12;21) translocations in Guthrie cards (Table 2) suggest a different scenario. Thus, if t(12;21) carrying cells demonstrated in Guthrie card analysis were fully leukaemic, their presence would imply a tumour burden of 1 : 10^5^ to 1 : 10^6^, corresponding to the number of nucleated cells captured in a Guthrie card blood spot (Gale *et al*, 1997; Taub *et al*, 2002). However, MRD studies of childhood ALL have demonstrated that a tumour burden of 1 : 10^5^ after a few months of therapy is associated with a high risk of relapse even during therapy (van Dongen *et al*, 1998), which is hardly compatible with the neonatal presence of t(12;21) in children diagnosed with leukaemia at ages of nearly 6 years. Instead, t(12;21) carrying cells present at birth most likely represent clones of leukaemia-predisposed cells which need to acquire more genetic aberrations to become fully malignant. A rather low concordance of childhood ALL in monozygotic twins would be consistent with this hypothesis (Ford *et al*, 1998), as would the demonstration of identical TEL-AML1 fusion genes in a set of monozygotic twins diagnosed with t(12;21) B-precursor ALL at ages 9 and 14 years (Wiemels *et al*, 1999b) as well as the simultaneous existence of identical TEL-AML1 fusion genes and non-identical deletions of the TEL gene, also believed to play a role in leukaemia development, in a pair of monozygotic twins concordant for t(12;21) positive ALL (Maia *et al*, 2001). In addition, a recent published paper indicated that t(12;21)-positive cells may be demonstrated in as many as one in 100 newborns, i.e. 100 times the proportion of children that will develop overt t(12;21)-positive ALL before the age of 15 years (Mori *et al*, 2002).

In conclusion, we demonstrated the presence of cells carrying leukaemia-specific TEL-AML1 fusion genes in the Guthrie cards of three patients diagnosed with B-precursor ALL at ages up to nearly 6 years. Our findings provide further evidence that the development of this type of leukaemia may be initiated *in utero* and are consistent with the hypothesis that ALL in childhood develop as a multiple hit process involving both pre- and postnatal genetic events.

## References

[bib1] AndersenMTNordentoftIHjalgrimLLChristiansenCLJakobsenVDHjalgrimHPallisgaardNMadsenHOChristiansenMRyderLPClausenNHoklandPSchmiegelowKMelbyeMJorgensenP2001 Characterization of t(12;21) breakpoint junctions in acute lymphoblastic leukemia Leukemia 15 858859 11368451 10.1038/sj.leu.2402095

[bib2] FaschingKPanzerSHaasOAMarschalekRGadnerHPanzer-GrumayerER2000 Presence of clone-specific antigen receptor gene rearrangements at birth indicates an in utero origin of diverse types of early childhood acute lymphoblastic leukemia Blood 95 27222724 10753857

[bib3] FordAMBennettCAPriceCMBruinMCVan WeringERGreavesM1998 Fetal origins of the TEL-AML1 fusion gene in identical twins with leukemia Proc Natl Acad Sci USA 95 45844588 9539781 10.1073/pnas.95.8.4584 PMC22533

[bib4] GaleKBFordAMReppRBorkhardtAKellerCEdenOBGreavesMF1997 Backtracking leukemia to birth: identification of clonotypic gene fusion sequences in neonatal blood spots Proc Natl Acad Sci USA 94 1395013954 9391133 10.1073/pnas.94.25.13950 PMC28413

[bib5] GreavesM1999 Molecular genetics, natural history and the demise of childhood leukaemia Eur J Cancer 35 173185 10448256 10.1016/s0959-8049(98)00433-x

[bib6] GreavesM2002 Childhood leukaemia BMJ 324 283287 11823363 10.1136/bmj.324.7332.283 PMC1122200

[bib7] LarsenLAChristiansenMNorgaard-PedersenBVuustJ1996 Quantitative detection of male DNA by polymerase chain reaction using a single primer set: application to sex determination and counting of rare fetal cells Anal Biochem 240 148150 8811895 10.1006/abio.1996.0342

[bib8] MaiaATFordAMJalaliGRHarrisonCJTaylorGMEdenOBGreavesMF2001 Molecular tracking of leukemogenesis in a triplet pregnancy Blood 98 478482 11435320 10.1182/blood.v98.2.478

[bib9] McCabeERHuangSZSeltzerWKLawML1987 DNA microextraction from dried blood spots on filter paper blotters: potential applications to newborn screening Hum Genet 75 213216 3030923 10.1007/BF00281061

[bib10] MoriHColmanSMXiaoZFordAMHealyLEDonaldsonCHowsJMNavarreteCGreavesM2002 Chromosome translocations and covert leukemic clones are generated during normal fetal development Proc Natl Acad Sci USA 99 82428247 12048236 10.1073/pnas.112218799 PMC123052

[bib11] Panzer-GrumayerERFaschingKPanzerSHettingerKSchmittKStockler-IpsirogluSHaasOA2002 Nondisjunction of chromosomes leading to hyperdiploid childhood B-cell precursor acute lymphoblastic leukemia is an early event during leukemogenesis Blood 100 347349 12070048 10.1182/blood-2002-01-0144

[bib12] RomanaSPPoirelHLeconiatMFlexorMAMauchauffeMJonveauxPMacintyreEABergerRBernardOA1995 High frequency of t(12;21) in childhood B-lineage acute lymphoblastic leukemia Blood 86 42634269 7492786

[bib13] RubnitzJELookAT1998 Molecular genetics of childhood leukemias J Pediatr Hematol Oncol 20 111 9482406 10.1097/00043426-199801000-00001

[bib14] SimonsenHBrandtNJNorgaard-PedersenB1998 (Neonatal screening in Denmark. Status and future perspectives) Ugeskr Laeger 160 57775782 9782755

[bib15] StormHHMichelsenEVClemmensenIHPihlJ1997 The Danish Cancer Registry–history, content, quality and use Dan Med Bull 44 535539 9408738

[bib16] TaubJWKonradMAGeYNaberJMScottJSMatherlyLHRavindranathY2002 High frequency of leukemic clones in newborn screening blood samples of children with B-precursor acute lymphoblastic leukemia Blood 99 29922996 11929791 10.1182/blood.v99.8.2992

[bib17] TrkaJZunaJZavacka-polouckovaAMadzoJHolzelovaEBrabencovaAKalinovaMZorneroveTHorakJZemanovaZHrusakO2000 Evidence for the presence of t(12;21) in cord blood samples of healthy newbornsProceedings of the 42nd meeting of The American Society of Haematology

[bib18] van DongenJJSeriuTPanzer-GrumayerERBiondiAPongers-WillemseMJCorralLStolzFSchrappeMMaseraGKampsWAGadnerHVan WeringERLudwigWDBassoGde BruijnMACazzanigaGHettingerKvan der Does-van den BergAHopWCRiehmHBartramCR1998 Prognostic value of minimal residual disease in acute lymphoblastic leukaemia in childhood Lancet 352 17311738 9848348 10.1016/S0140-6736(98)04058-6

[bib19] WiemelsJLCazzanigaGDaniottiMEdenOBAddisonGMMaseraGSahaVBiondiAGreavesMF1999a Prenatal origin of acute lymphoblastic leukaemia in children(see comments)Lancet 354 14991503 10551495 10.1016/s0140-6736(99)09403-9

[bib20] WiemelsJLFordAMVan WeringERPostmaAGreavesM1999b Protracted and variable latency of acute lymphoblastic leukemia after TEL-AML1 gene fusion in utero Blood 94 10571062 10419898

[bib21] WiemelsJLXiaoZBufflerPAMaiaATMaXDicksBMSmithMTZhangLFeusnerJWienckeJPritchard-JonesKKempskiHGreavesM2002 In utero origin of t(8;21) AML1-ETO translocations in childhood acute myeloid leukemia Blood 99 38013805 11986239 10.1182/blood.v99.10.3801

[bib22] YagiTHibiSTabataYKuriyamaKTeramuraTHashidaTShimizuYTakimotoTTodoSSawadaTImashukuS2000 Detection of clonotypic IGH and TCR rearrangements in the neonatal blood spots of infants and children with B-cell precursor acute lymphoblastic leukemia Blood 96 264268 10891460

